# Immune-related transcripts, microbiota and vector competence differ in dengue-2 virus-infected geographically distinct *Aedes aegypti* populations

**DOI:** 10.1186/s13071-023-05784-3

**Published:** 2023-05-19

**Authors:** Tse-Yu Chen, Jovana Bozic, Derrick Mathias, Chelsea T. Smartt

**Affiliations:** 1 Florida Medical Entomology Laboratory, Department of Entomology and Nematology, University of Florida, Vero Beach, FL USA; 2 Section of Infectious Diseases, Department of Internal Medicine, Yale School of Medicine, Yale University, New Haven, CT USA; 3 Department of Entomology, The Center for Infectious Disease Dynamics, The Huck Institutes of the Life Sciences, Pennsylvania State University, University Park, PA USA

**Keywords:** Vector competence, DENV-2, *Aedes aegypti*, Immune response, Microbiota

## Abstract

**Background:**

Vector competence in *Aedes aegypti* is influenced by various factors. Crucial new control methods can be developed by recognizing which factors affect virus and mosquito interactions.

**Methods:**

In the present study we used three geographically distinct *Ae. aegypti* populations and compared their susceptibility to infection by dengue virus serotype 2 (DENV-2). To identify any differences among the three mosquito populations, we evaluated expression levels of immune-related genes and assessed the presence of microbiota that might contribute to the uniqueness in their vector competence.

**Results:**

Based on the results from the DENV-2 competence study, we categorized the three geographically distinct *Ae. aegypti* populations into a refractory population (Vilas do Atlântico), a susceptible population (Vero) and a susceptible but low transmission population (California). The immune-related transcripts were highly expressed in the California population but not in the refractory population. However, the Rel-1 gene was upregulated in the Vilas do Atlântico population following ingestion of a non-infectious blood meal, suggesting the gene’s involvement in non-viral responses, such as response to microbiota. Screening of the bacteria, fungi and flaviviruses revealed differences between populations, and any of these could be one of the factors that interfere with the vector competence.

**Conclusions:**

The results reveal potential factors that might impact the virus and mosquito interaction, as well as influence the *Ae. aegypti* refractory phenotype.

**Graphical Abstract:**

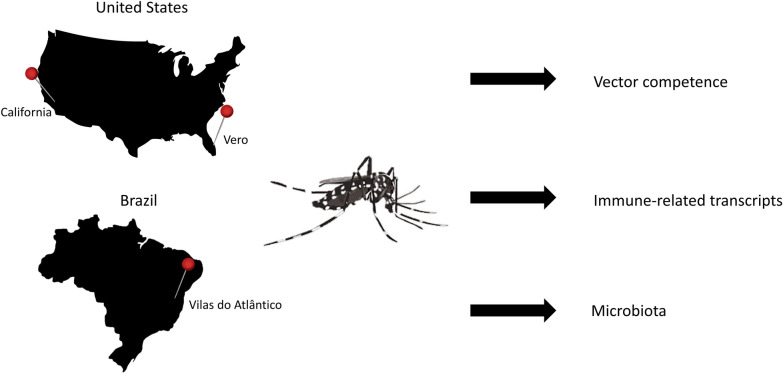

**Supplementary Information:**

The online version contains supplementary material available at 10.1186/s13071-023-05784-3.

## Background

The four serotypes of dengue virus (DENV1–4;* Flaviviridae*;* Flavivirus*) are a major health risk for people living in tropical and subtropical areas, and every year 50–100 million individuals suffer symptomatic infections [1, 2]. Current models of the risk for DENV infection predict an even greater annual exposure in the upcoming 30–60 years [3]. DENV is a mosquito-borne virus transmitted by vector species in the genus *Aedes*, with the principal vector being *Aedes aegypti* [4]. Increased international travel and trade has been a major factor driving the successful expansion of *Ae. aegypti* from its native areas into new, formerly uninhabited ones. Nowadays, *Ae. aegypti* populations can be found in the Americas, Europe, Africa, Asia and Oceania. Climate change is expected to contribute to a further expansion of the global distribution of these populations [5].

For mosquito-borne viruses, vector competence is defined as the ability of a mosquito to serve as a disease vector, and encompasses midgut infection, dissemination to other tissues, and transmission of the virus through saliva [6]. For a mosquito to become infectious, the virus must pass several barriers, including the midgut infection barrier (MIB), midgut escape barrier (MEB), salivary gland infection barrier (SGIB) and salivary gland escape barrier (SGEB) [7]. The interval between acquisition of virus by a vector and the vector becoming capable of transmission is called the extrinsic incubation period (EIP) [8]. The EIP is an important parameter that influences vectorial capacity and has been shown to vary among populations infected with flaviviruses, such as yellow fever virus (YFV), DENV and Zika virus (ZIKV), as well as with alphaviruses, such as chikungunya virus (CHIKV) [9].

The mosquito innate immune response is one of the important factors that can influence vector competence [6]. The RNA interference (RNAi) pathway is considered to be the main antiviral pathway in mosquitoes and to be involved in the degradation of viral RNA [10–13]. Antiviral activity is also regulated via several innate immune pathways [14], including the Toll pathway [15, 16], the immune deficiency (IMD) pathway [17, 18] and the Janus kinase/signal transducer and activator of transcription (JAK/STAT) pathway [19, 20]. These three major pathways involve pattern recognition followed by signal transduction to trigger the expression of downstream antimicrobial peptides or other immune factors. Other immune pathways might also participate in the antiviral response, such as apoptosis [21, 22] and autophagy [22, 23].

Mosquito microbiota, including bacteria, fungi and insect-specific viruses (ISVs), can influence a range of host phenotypes, including vector competence [24–29]. For example, a number of studies have shown that bacteria in *Ae. aegypti* can influence DENV infection [16, 30–32]. Moreover, both insect-specific viruses and fungi can also impact vector competence and alter virus infection dynamics within the mosquito [27, 33–35]. The microbiota is diverse within and between mosquito populations [24] and varies in mosquito strains that are differentially susceptible to viruses [36].

In the present study, we compared three geographically distinct *Ae. aegypti* populations for their susceptibility to infection and ability to transmit DENV-2. To identify potential factors responsible for differences in vector competence, we focused on each of the major immune pathways, selecting key genes in three categories to represent pathways. These included: (i) transcription factors, such as Rel-1A (Toll), Rel-1B (Toll), Rel-2 (IMD); (ii) receptors, such as Dome (JAK/STAT); or (iii) effectors, such as ATG5 (autophagy), Ago-2 (RNAi), Dronc (apoptosis) and IAP-1 (apoptosis). We then characterized their transcript levels after infection with DENV serotype 2 (DENV-2) infection during the first 3 days of the extrinsic incubation period and compared expression profiles among populations. To examine additional factors that may contribute to variation in vector competence, we screened bacterial, fungal, and viral components of the microbiota by PCR. We conclude by discussing the potential connection of variation in the microbiota and innate immunity to population-level differences in DENV-2 infection dynamics within the mosquito.

## Methods

### Mosquitoes

Eggs of *Ae. aegypti* were collected from 2015 to 2017 at locations in the Metropolitan Region of Salvador (Vilas do Atlântico area) in Bahia State, Brazil using standard oviposition traps; this population is hereafter referred to as the Vilas do Atlântico population. Mosquito eggs were also collected from California (in 2016) and from Vero Beach, Florida, USA (in 2015). These two North American populations were used in this study and are hereafter referred to as the California population and the Vero population, respectively. *Aedes aegypti* eggs were reared at 28 °C and 60–80% relative humidity in a climate-controlled room under a light:dark cycle of 14:10 h. After hatching, larvae were separated into pans at an approximate density of 200 larvae per pan and provided with 3 ml of larval food (5 g brewer’s yeast and 5 g liver powder mixed with 1 L water) daily. Adults were provided 20% sucrose solution-soaked cotton rolls ad libitum. Mosquito colonies were maintained by feeding female mosquitoes on blood from chickens following approved standard protocols (IACUC protocol 201807682) [37, 38]. The Vilas do Atlântico F4, California F7 and Vero F8 generations were used in the study.

### Infection of *Ae. aegypti*

*Aedes aegypti* females aged 4–6 days were placed into two groups (control and infected). The control group contained 150 females from each population fed on defibrinated bovine blood (Hemostat, Dixon, CA, USA) supplemented with 1 mM adenosine triphosphate (ATP; Thermo Fisher Scientific, Waltham, MA, USA) without virus. The infection group comprised 250 females that were fed on defibrinated bovine blood supplemented with 1 mM ATP and containing DENV-2 (New Guinea C strain; GenBank accession #KM204118). The virus was inoculated into African green monkey (Vero) cells at a multiplicity of infection of 0.1 viruses per cell and incubated for 5 days at 37 °C supplemented with 5% CO_2_. One day before infection, female mosquitoes were transferred to 16-oz paperboard carton boxes (WebstaurantStore, Lancaster, PA, USA; 50 female mosquitoes/carton box) and the boxes placed in an incubator under the same conditions as the climate-controlled room. Sugar water was removed 24 h before blood-feeding. The defibrinated bovine blood (Hemostat) mixed either with supernatant from fresh virus culture or with medium from the 5-day-old culture of Vero cells without virus and supplemented with 1 mM ATP was supplied to mosquitoes using an artificial feeding apparatus (Hemotek Ltd., Great Harwood, Blackburn, UK) for 1 h of feeding. The ratio of blood to medium with or without virus was 2:1. After feeding, fully blood-engorged mosquitoes were collected into carton boxes and provided with 20% sucrose solution ad libitum. Freshly fed, three fully blood-engorged mosquitoes in each population and a blood sample from the assay were collected immediately following feeding for determining virus titer. Female mosquitoes were collected from the control and infected groups at 1, 2, and 3 days post-infection (dpi) in four biological replicates of five mosquitoes per replicate and placed at − 80 °C. The same infected mosquito pools collected at 1, 2 and 3 dpi were also used to measure the virus titer. On 10 dpi, the remaining mosquitoes in each population were collected and separated into bodies and legs. Saliva was collected after the legs were removed but before the bodies were separated out using the capillary method following well-established protocols [39]. All samples were placed at − 80 °C for subsequent RNA extraction.

### RNA extraction

TRIzol Reagent (Invitrogen, Thermo Fisher Scientific, Waltham, MA, USA) was used to extract RNA from all mosquito samples as previously described [39]. Whole mosquito bodies were collected individually for vector competence estimation and in pools of five after 1, 2, 3 dpi for gene expression, and RNA was extracted following the same protocol as described in following text. At 10 dpi, RNA from individual mosquito bodies, legs, and saliva was used to estimate virus titers and determine infection rates (number of mosquito bodies containing viral RNA divided by the number of blood engorged mosquitoes), dissemination rates (number of mosquito legs containing viral RNA divided by the number of mosquito bodies containing viral RNA), and transmission rates (number of mosquito saliva samples containing viral RNA divided by the number of mosquito legs containing viral RNA), respectively. Body and leg samples were placed in 1.5-ml Eppendorf tubes with 0.2 ml TRIzol and ten 2-mm-diameter glass beads (Thermo Fisher Scientific) and homogenized using a bullet blender tissue homogenizer (Next Advance, Troy, NY, USA). After homogenization, standard RNA extraction was completed following well-established protocols [38, 40]. Briefly, 0.3 ml TRIzol was added to the homogenized tissue solution and incubated for 10 min, following which 0.1 ml of chloroform was added to the solution. The solution was then incubated for 15 min and subsequently centrifuged, and the aqueous phase was collected and mixed with 0.25 ml isopropanol. Following centrifugation of the aqueous for 10 min, the supernatant was discarded, and the pellet was washed in 70% ethanol and resuspended into DEPC-treated water. Saliva samples preserved in 0.1 ml phosphate-buffered saline (pH 7.4) solution were extracted following the RNA extraction protocol described above, without homogenization. All RNA samples were stored at − 80 °C.

### Reverse transcription and real-time PCR

All RNA samples were quantified on a Nanodrop 2000 spectrophotometer (Thermo Fisher Scientific). RNA was treated with RQ1 RNase-Free DNase (Promega, Madison, WI, USA) to degrade any DNA carried over from the RNA extraction. For the samples used to examine gene expression, Enhanced Avian Reverse Transcriptase (Sigma-Aldrich, St. Louis, MO, USA) was used for reverse transcription, and oligo dT primer was used to generate copy DNA (cDNA) following standard protocols. Gene expression was characterized on a CFX96™ real-time thermal cycler (Bio-Rad Laboratories, Hercules, CA, USA) using the SsoAdvanced SYBR Green Supermix Kit (Bio-Rad Laboratories) and specific primer sets designed to amplify target genes in the mosquito innate immune pathways [23, 38], with 100 ng of cDNA per sample (Additional file 1: Table S1). *Aedes aegypti* ribosomal protein S7 gene (GenBank Accession #AY380336) was used as a control for standardizing and the 2^-ΔΔCt^ (delta-delta Ct) method was applied for calculating relative gene expression values [39]. All samples were duplexed and followed well-established protocols [40, 41]. In the virus titer quantification experiment, the virus genome equivalents were estimated by quantitative reverse transcription (RT)-PCR standardized with plaque assay as described previously [42]. In brief, RNA samples were quantified and detected on a CFX96™ real-time thermal cycler (Bio-Rad Laboratories) using the iTaq Universal One-Step RT-qPCR Kits (Bio-Rad Laboratories). The values were applied into the regression line built from the plaque assay and the log10 plaque-forming unit equivalents (PFUe) of DENV-2/ml (log PFUe/ml) were estimated.

In the flavivirus ISV screening study, 100 ng DNase-treated RNA from uninfected mosquitoes was combined with flavivirus universal primers at the concentration used in a previous study [43] and reagents from the iTaq Universal One-Step RT-qPCR Kit (Bio-Rad Laboratories) and analyzed on the CFX96™ Real-Time PCR thermal cycler (Bio-Rad Laboratories). After the reaction, positive samples were analyzed by gel electrophoresis in a 1% agarose gel. Distinct bands of the expected length were excised, and PCR amplicons were extracted using the GenElute Gel Extraction Kit (Sigma-Aldrich). A second PCR reaction was conducted with DreamTaq DNA Polymerase (Thermo Fisher Scientific) using the same flavivirus primer set to generate a sufficient amount of DNA for cloning. PCR conditions consisted of 1 cycle of 95 °C for 3 min, then 35 cycles of 95 °C for 30 s, 60 °C for 30 s, and 72 °C for 1 min, followed by 1 cycle of 72 °C for 5 min. The PCR products were cleaned up using the GenElute Gel Extraction Kit (Sigma-Aldrich) as described by the manufacturer and placed at − 20 °C for cloning.

### DNA extraction and cloning of microbial PCR amplicons

To examine variation among the three mosquito populations for bacterial/fungal microbiota, three pools of three blood-fed female mosquitoes per population were collected at 2 days post-feeding. DNA was extracted from each pool using the E.Z.N.A. Tissue DNA kit (Omega Bio-Tek, Norcross, GA, USA) and stored at − 80 °C until use. The DNA samples were used as templates for PCRs targeting bacterial 16S ribosomal RNA (rRNA) [44] and fungal 18S rRNA genes [45]. These PCR products, as well as those from the flavivirus PCR described above, were cloned (CloneJET PCR Cloning Kit; Thermo Fisher Scientific) into NEB 5-alpha Competent* Escherichia coli* (New England Biolabs, Ipswich, MA, USA) and grown on selective LB agar plates with carbenicillin (100 μg/ml). All the colonies from each sample were picked for both 16S and 18S to screen for sequence variation using restriction fragment length polymorphism (RFLP) analysis [46]. Briefly, plasmid-specific primers (pJET1.2 Forward and Reverse) were used to amplify DNA inserts, after which the amplicons were digested with restriction enzymes* Alu*I and* Hha*I (New England Biolabs) for fungi, and with* Hpa*II,* Cla*I and* Hin*P1I (New England Biolabs) for bacteria, and then electrophoresed in 1% agarose gels. Differentiating amplicon variants or RFLP patterns of 16S rRNA bacterial and 18S rRNA fungal PCR products were obtained. Colonies that had unique banding patterns were grown overnight in LB liquid media with carbenicillin (100 μg/ml). Plasmid DNA was purified using the GeneJET Plasmid Miniprep Kit (Thermo Fisher Scientific), quantified spectrophotometrically and submitted for Sanger sequencing (Eurofins Genomics, Louisville, KY, USA). Flavivirus colonies were selected randomly from each population and were not subjected to RFLP analysis. The bacterial/fungal and flavivirus operational taxonomic units (OTUs) were then identified by BLAST via the NCBI database.

### Statistical analysis

Fisher’s exact test was used to compare rates of feeding, infection, dissemination and transmission in the vector competence study. Virus titer was analyzed in freshly fed mosquitoes and in mosquitoes at 10 dpi, using the Kruskal–Wallis test and Dunn's test for multiple comparisons. To compare Toll pathway gene transcript levels after blood-feeding and virus titer in the first 3 days of the incubation period between populations, we used two-way analysis of variance (Two-Way ANOVA) with time point and population as independent variables, followed by Tukey's post hoc test for multiple pairwise comparisons. Transcript levels of *Ae. aegypti* immune pathway genes were compared to the control group using the Wilcoxon test. All statistical analyses were performed using JMP Pro (www.jmp.com) and GraphPad Prism 9 (www.graphpad.com). The figures were made with GraphPad Prism 9 (GraphPad Software, San Diego, CA, USA).

## Results

### Estimation of vector competence in the different *Ae. aegypti* populations under DENV-2 infection

Vector competence studies were conducted at 10 dpi with the three *Ae. aegypti* populations from the Americas: Vilas do Atlântico (VDA), California (CA), and Vero. All populations were fed with blood containing 7.23 ± 0.62 log PFUe/ml of DENV-2. Freshly fed VDA, CA and Vero mosquitoes imbibed a mean (± standard deviation [SD]) of 4.29 ± 0.43, 4.23 ± 0.5 and 4.03 ± 0.53 log PFUe/ml of DENV-2, respectively. The virus titers in blood meals did not significantly differ between populations (Kruskal–Wallis test, *P* > 0.05). The feeding rates of controls for the VDA, CA and Vero populations were 74.7% (112/150), 66.7% (100/150) and 81% (81/100), respectively, while the feeding rates of these populations on virus-containing blood were 38.4% (93/250), 44% (107/250) and 54.4% (133/250), respectively. The CA population had lower blood-feeding rates on non-DENV-2-containing blood than the Vero population (Fisher’s exact test,* P* = 0.014), and the Vero population had higher feeding rates with DENV-2-containing blood than the VDA (Fisher’s exact test, *P* < 0.001) and CA (Fisher’s exact test, *P* = 0.03) populations.

The infection rate varied among the three populations tested, from a low of 50% in the VDA population (*n* = 18), increasing to 72.4% in the CA population (*n* = 29) and 82.8% in the Vero population (*n* = 29) (Table 1). The two populations from North America, CA, and Vero, had higher DENV-2 infection rates than the population from South America, VDA, but differences were only significant between VDA and Vero (Fisher’s exact test, *P* = 0.024).

**Table 1:** Infection, dissemination and transmission rates of the three *Aedes aegypti* populations at 10 days post-oral infection with dengue virus serotype 2

Mosquito population	Number tested	Infection rate	Dissemination rate	Transmission rate	Transmission efficiency
Vilas do Atlântico (VDA)	18	9 (50.0%)^a^	4 (44.4%)^c^	1 (25.0%)^e,f^	5.6%^g^
California (CA)	29	21 (72.4%)^a,b^	17 (81.0%)^c,d^	5 (29.4%)^e^	17.2%^g^
Vero	29	24 (82.8%)^b^	21 (87.5%)^d^	15 (71.4%)^f^	51.7%^h^

Values followed by different lowercase letters are significantly different at *P* < 0.05

The dissemination rate in the VDA population was 44.4% (*n* = 9), while the CA and Vero populations had dissemination rates of 81.01% (*n* = 21) and 87.5% (*n* = 24), respectively (Table 1). Although the CA and Vero populations had higher dissemination rates than VDA, similar to the infection rates, the only significant difference was between the Vero and VDA populations (Fisher’s exact test, *P* = 0.02).

For the transmission rate, the data indicated rates of 25% (*n* = 4), 29.4% (*n* = 17) and 71.4% (*n* = 21) in the VDA, CA and Vero populations, respectively (Table 1). The Vero population had the highest transmission rate, which was significantly greater than the transmission rate of the CA population (Fisher’s exact test, *P* = 0.028).

The transmission efficiency (TE) (number of mosquito saliva samples containing viral RNA divided by the number of blood-fed mosquitoes) was 5.6%, 17.2% and 51.7% in the VDA, CA and Vero populations, respectively (Table 1). The Vero population had the highest TE and was significantly different to the TE in the VDA (Fisher’s exact test, *P* = 0.001) and CA (Fisher’s exact test, *P* = 0.01) populations.

**Fig. 1:**
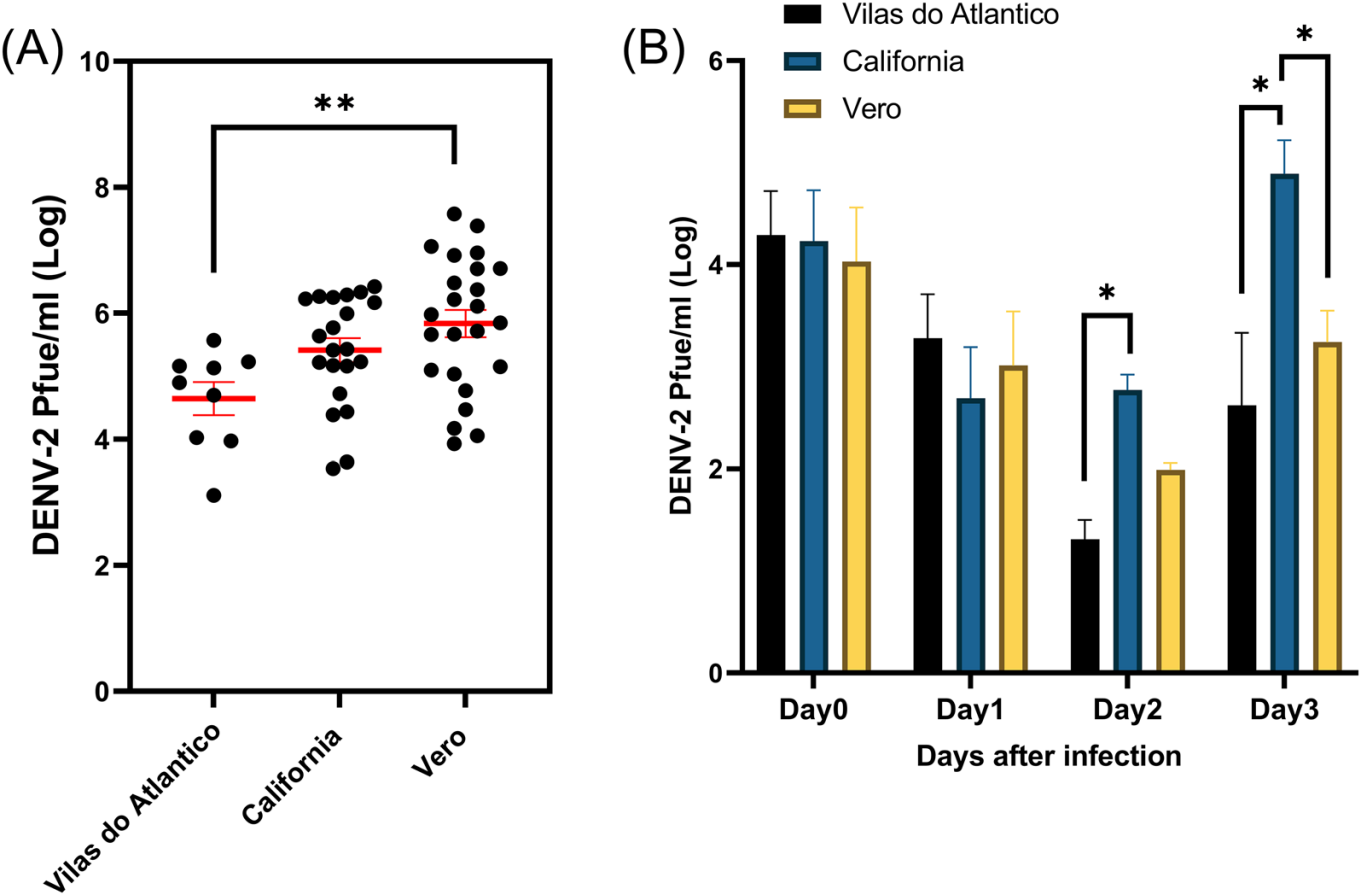
Variations in DENV-2 titer among the three mosquito populations Vilas do Atlântico (VDA), California (CA) and Vero. **A** DENV-2 titer at 10 days post-acquisition of a DENV-2-containing blood meal. Each data point represents a virus-infected individual. Bars represent standard error of the mean. **B** Mean DENV-2 titers among the VDA, CA and Vero mosquito populations at days 1, 2 and 3 post-infectious blood meal. Bar represents standard deviation of the mean. Asterisks indicate a statistical difference at *P < 0.05 and **P < 0.01. DENV-2, Dengue virus serotype 2; Pfue, plaque-forming unit equivalents

Mean (± SD) titers of DENV-2 at 10 dpi in mosquito body samples were 5.32 ± 0.45, 5.41 ± 0.85 and 5.83 ± 1.04 log PFUe/ml in the VDA, CA, and Vero populations, respectively (Fig. 1a). The VDA population had the lowest mean DENV-2 titer, which was significantly different from the mean DENV-2 titer in the Vero population (Kruskal–Wallis test, *P* = 0.009), but not that in the CA population (Kruskal–Wallis test, *P* = 0.13).

### Detection of DENV-2 titer in the early infection period

DENV-2 titer was determined to ensure virus replication in each population at the first 3 dpi. The eclipse phase occurred in all three populations; DENV-2 titer decreased at 1 dpi compared to the titer at the time point of the infectious blood meal (day 0, Fig. 1b). The VDA, CA, and Vero populations had mean (± SD) DENV-2 titers of 3.28 ± 0.19, 2.69 ± 0.15 and 3.01 ± 0.07 log PFUe/ml, respectively. The DENV-2 titer continued to decrease at 2 dpi in the VDA (1.31 ± 0.71 log PFUe/ml) and Vero (1.99 ± 0.31 log PFUe/ml) populations but not in the CA (2.77 ± 0.33 log PFUe/ml) population. Compared to the VDA and Vero populations, the CA population had a relatively higher titer at 2 dpi, which was significantly different from the titer in the VDA population (Two-Way ANOVA, *P* = 0.005). At 3 dpi, DENV-2 titers increased in all populations, with the VDA, CA and Vero populations having titers of 2.62 ± 0.33, 4.88 ± 0.16 and 3.24 ± 0.79 log PFUe/ml), respectively. The CA population had the highest DENV-2 titer at 3 dpi, which was significantly different from the titers in the Vero (Two-Way ANOVA, *P* = 0.001) and VDA (Two-Way ANOVA, *P* < 0.0001) populations, with the VDA population having the lowest DENV-2 titer.

### Alterations in transcript level of *Ae. aegypti* immune pathway genes early in the DENV-2 infection period

**Fig. 2:**
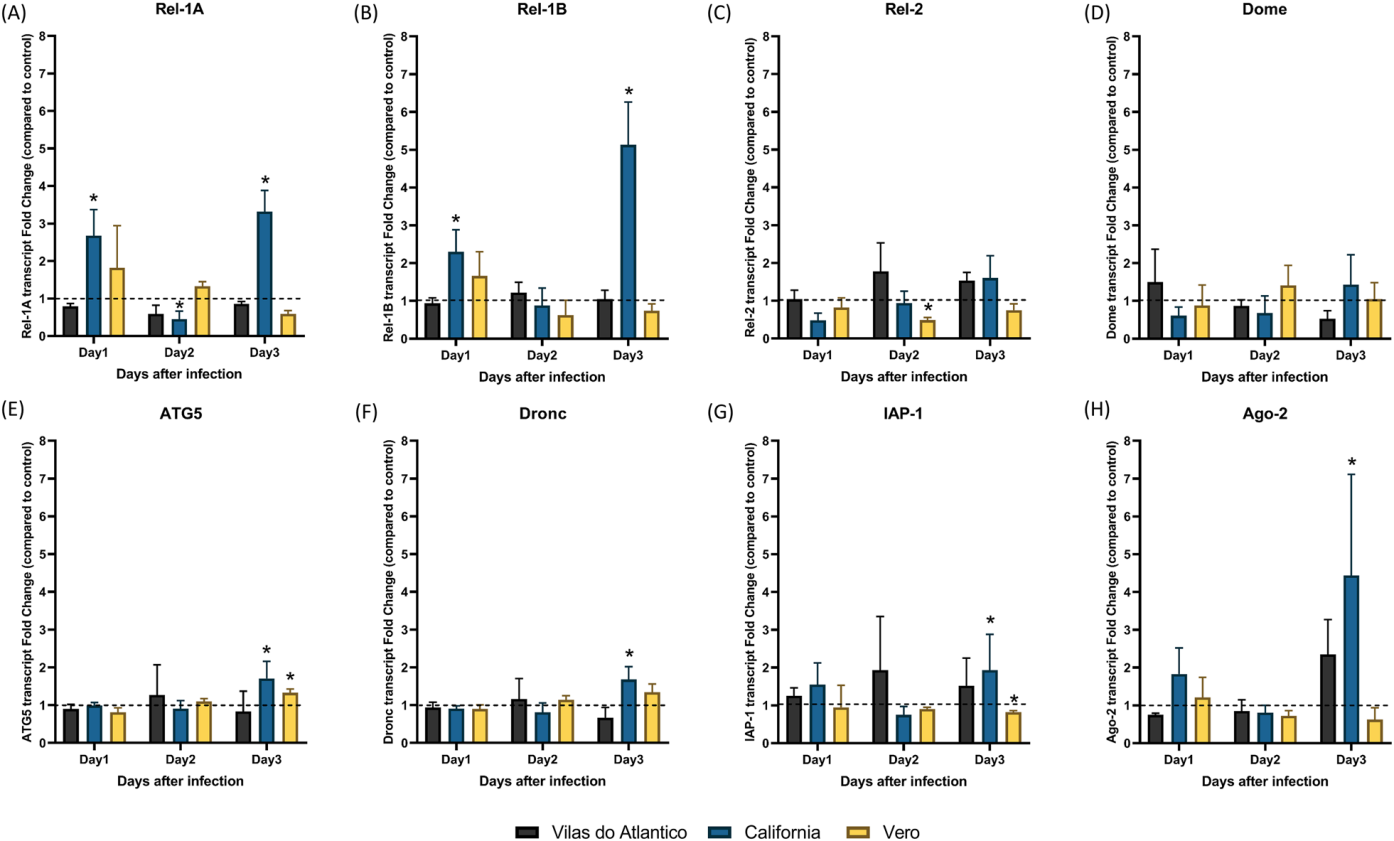
Relative fold change in immune pathway-related transcripts during the first 3 days post-feeding between the virus-infected group and blood-only group in the three *Aedes aegypti* populations Vilas do Atlântico, California and Vero.** A**-**H** Mean relative transcript levels of Rel-1A (**A**), Rel-1B (**B**), Rel-2 (**c**), Dome (**D**), ATG5 (**E**), Dronc (**F**), IAP-1 (**G**), Ago-2 (**H**). Relative transcript values were calculated and standardized with the *Ae. aegypti* ribosomal protein S7 gene. Dashed line represents the baseline value (1.0) of the respective controls. Bars represent standard deviation of the mean. Asterisk indicates a statistical difference between blood-fed and DENV-2 virus-infected mosquitoes at *P < 0.05 according to the Wilcoxon test

The transcripts of several *Ae. aegypti* genes from multiple immune pathways were quantified during the first 3 days after DENV-2 infection. Compared to the blood-only control groups, mosquitoes fed with DENV-2-supplemented blood showed a variation in immune transcript levels among populations and time points (Fig. 2).

In the Toll pathway, Rel-1A, which functions as a transcription factor, did not show significant differences between DENV-2-infected mosquitoes and blood only-fed mosquitoes from the VDA and Vero populations. However, in the CA population, the mean (± SD) Rel-1A transcript level was levels at 1 dpi (2.68 ± 0.68, Wilcoxon test, *P* = 0.02) and 3 dpi (3.32 ± 0.56, Wilcoxon test, *P* = 0.049) but lower at 2 dpi (0.45 ± 0.21, Wilcoxon test, *P* = 0.043) (Fig. 2a). Rel-1B, another Toll pathway downstream transcription factor, showed a similar pattern, with the CA population having significantly higher transcript levels at 1 dpi (2.30 ± 0.58, Wilcoxon test, *P* = 0.021) and 3 dpi (5.13 ± 1.1, Wilcoxon test, *P* = 0.034) compared to the other populations (Fig. 2b).

The mean expression of Rel-2, a gene involved in the IMD pathway, was downregulated at 2 dpi in the Vero population (0.49 ± 0.07, Wilcoxon test, *p* = 0.021) but not in the other two mosquito populations (Fig. 2c). The transcript level of the JAK/STAT pathway receptor Dome did not vary after infection in any of the three mosquito populations (Fig. 2d). Autophagy related 5 (ATG5), which is involved in the extension of the phagophoric membrane in autophagic vesicles in the autophagy pathway, only showed a higher transcript level at 3 dpi in the CA population (1.71 ± 0.45, Wilcoxon test, *P* = 0.043) and Vero population (1.33 ± 0.10, Wilcoxon test, *P* = 0.021) (Fig. 2e). The apoptosis genes Dronc (1.68 ± 0.34, Wilcoxon test, *P* = 0.043) (Fig. 2f) and IAP-1 (1.93 ± 0.95, Wilcoxon test, *P* = 0.043) (Fig. 2g), and RNAi pathway gene Ago-2 (4.44 ± 2.67, Wilcoxon test, *P* = 0.042) (Fig. 2h) also showed significant increases in transcripts in the CA population at 3 dpi. In contrast, the transcription of IAP-1 (0.83 ± 0.04, Wilcoxon test, *P* = 0.004) was downregulated in the Vero population at 3 dpi.

### Toll pathway gene transcript levels between *Ae. aegypti* populations after blood-feeding

**Fig. 3:**
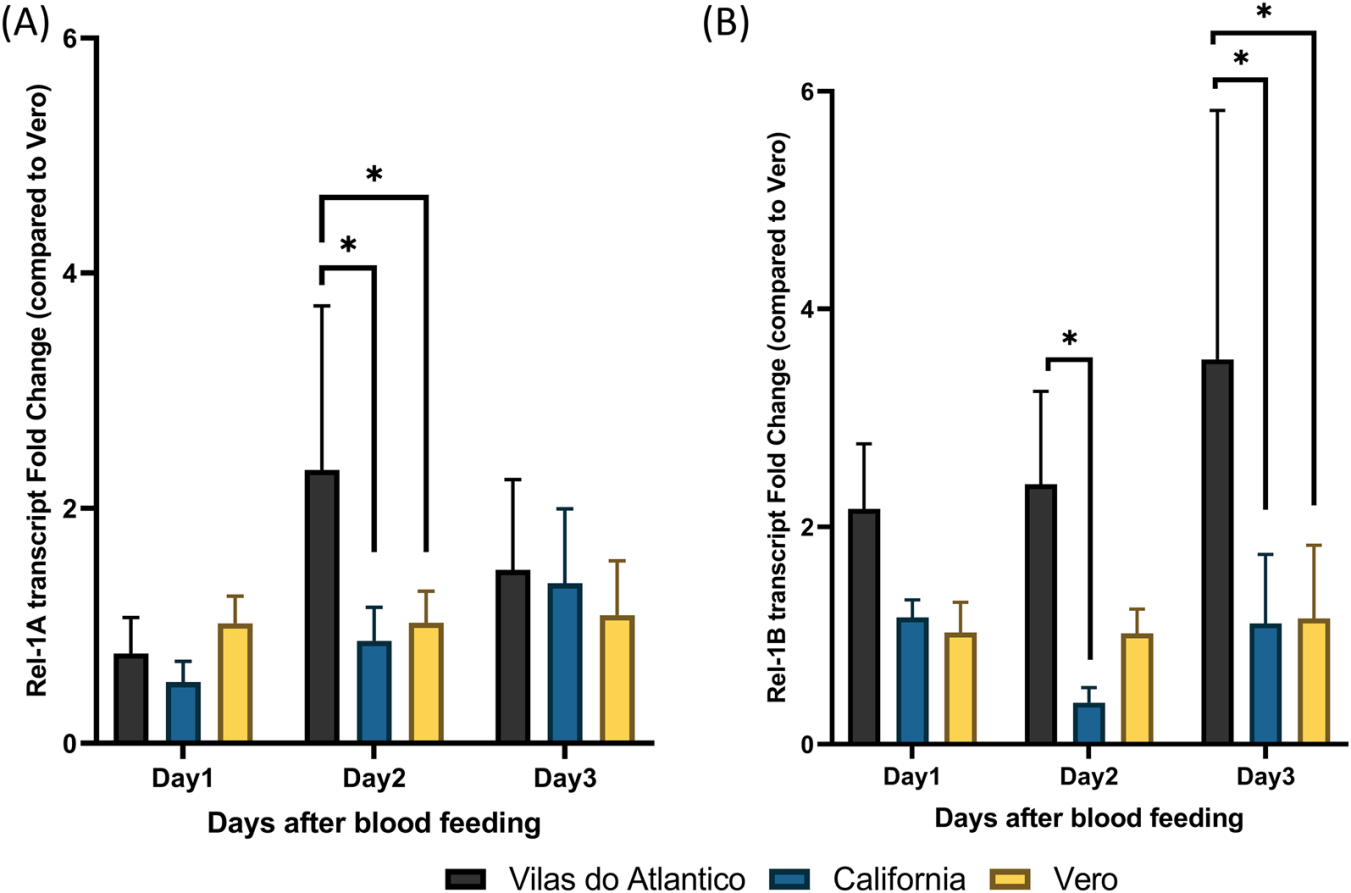
Relative Rel-1A and Rel-1B expression levels in the three *Aedes aegypti* populations Vilas do Atlântico, California and Vero at 1, 2, and 3 days following the acquisition of a non-infectious blood meal. **A** Mean value of Rel-1A expression fold change compared to the Vero population after blood-feeding. **B** Mean value of Rel-1B expression fold change compared to the Vero population after blood-feeding. Bars represent standard deviation of the mean. Asterisk indicates statistical difference at *P < 0.05

To examine population-level variation in genes involved in the immune system, we compared the transcript levels of selected immunity genes between the three populations after blood-feeding in the absence of DENV-2. Because the Vero population was the most susceptible to infection in this study, this population was used to normalize relative gene expression and to further calculate the differences in gene expression. Evaluation of the Toll pathway transcription factor Rel-1A revealed that the VDA population had the relatively highest level (mean ± SD: 2.33 ± 1.39), which was significantly different from that in the CA (0.87 ± 0.29, Two-Way ANOVA, *P* = 0.007) and Vero (1.03 ± 0.27, Two-Way ANOVA, *P* = 0.017) populations at 2 days post blood-feeding (dpb) (Fig. 3a). For another Toll pathway transcription factor, Rel-1B, the VDA population had significantly higher transcript levels at 2 dpb (2.39 ± 0.85) compared to the transcript levels in the CA population (0.38 ± 0.14, Two-Way ANOVA, *P* = 0.01). Moreover, at 3 dpb, the transcript level of Rel-1B remained the highest in the VDA population (3.53 ± 2.29) and was significantly higher than the transcript level in the CA population (1.11 ± 0.63, Two-Way ANOVA, *P* = 0.005) and Vero (1.16 ± 0.67, Two-Way ANOVA, *p* = 0.003) populations (Fig. 3b).

Transcript abundance of the Toll pathway genes, IMD pathway gene Rel-2 and JAK/STAT pathway gene Dome were assessed (Additional file 1: Table S2). The Rel-2 transcript was downregulated at 2 dpb in the CA population (0.39 ± 0.23) compared to the Vero population (1.01 ± 0.2, Two-Way ANOVA, *P* = 0.02). The Rel-2 gene was upregulated at 3 dpb in the CA population (3.92 ± 2.9) compared to Vero population (1.03 ± 0.31, Two-Way ANOVA, *P* = 0.018). The Dome transcript in CA population (5.45 ± 0.5) was significantly higher than that in the Vero population at 3 dpb (1.09 ± 0.57, Two-Way ANOVA, *P* = 0.04).

### Differences in microbiota between the three *Ae. aegypti* populations

**Fig. 4:**
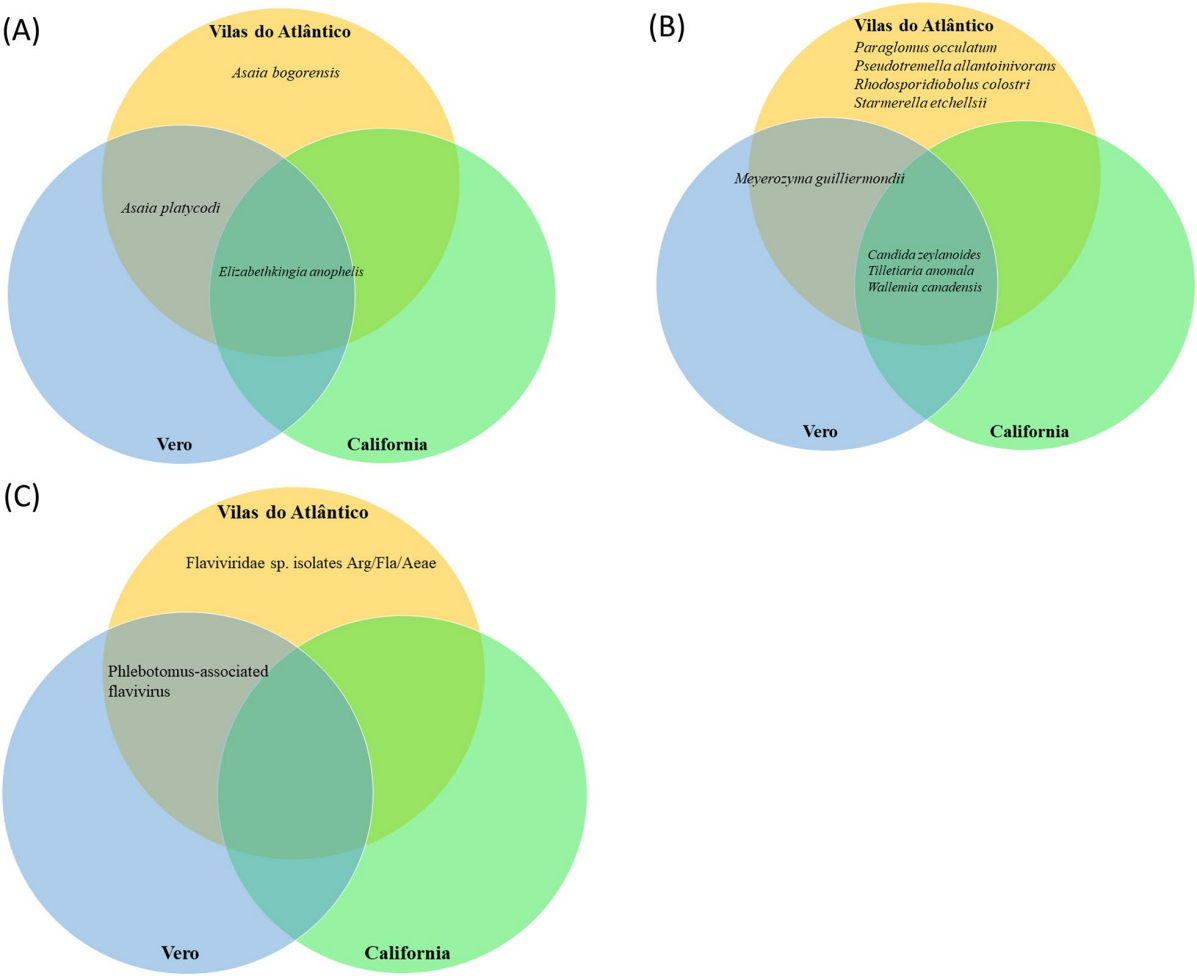
Venn diagram showing the microbiota in the three *Ae. aegypti* populations Vilas do Atlântico, California and Vero following blood-feeding. **A** Bacteria 16S rRNA library, **B** yeast/fungi 18S rRNA library, **C** flaviviruses NS5 library

Analysis of the 16S rRNA gene amplicons revealed that colonies cultured from mosquito samples were dominated by *Asaia bogorensis* and *Asaia platycodi* (*Alphaproteobacteria*) and by *Elizabethkingia anophelis* (*Flavobacteriia*) in the VDA population. *Asaia platycodi* and *E. anophelis* sequences were amplified from the Vero population as well, while the CA population produced sequences belonging only to the genus *Elizabethkingia* (Fig. 4a; Additional file 1: Tables S3, S4).

From fungal 18S rRNA gene libraries, we identified a total of eight species among all three mosquito populations. The VDA population generated the most diversity (Fig. 4b; Additional file 1: Tables S5, S6) with all eight fungal species identified, three of which were classified as the yeasts (*Sacharomycetes*) *Starmerella etchellsii*, *Candida zeylanoides* and *Meyerozyma guilliermondii*. Four species were discriminated as filamentous fungi (*Basidiomycetes*): *Pseudotremella allantoinivorans*, *Rhodosporidiobolus colostri*, *Tilletiaria anomala* and *Wallemia canadensis*. One species was categorized as mold-like fungi (*Mucoromycota*): *Paraglomus occulatum*. Four sequences were generated from the Vero population, including two yeast species (*C. zeylanoides*, and *M. guilliermondii*) and two filamentous fungi (*T. anomala* and *W. canadensis*). *Candida zeylanoides*, *T. anomala* and *W. canadensis* were the only species represented in fungal sequences identified from the CA population.

Flavivirus gene libraries were generated only from the VDA and Vero populations (Fig. 4c; Additional file 1: Table S7), with their sequences matching to previously described flavivirus isolates from *Ae. aegypti* in Argentina (*Flaviviridae* sp. isolates Arg/Fla/Aeae) and from a sand fly (*Phlebotomus* (*Phlebotomus*-associated flavivirus).

## Discussion

Vector competence is influenced by multiple factors, including the interaction between mosquito genotypes and microbiota [9]. *Aedes aegypti* populations from different geographic locations are highly diverse [47] and exhibit variability in DENV vector competence [9, 48]. To identify the factors associated with vector competence, it is critical to understand the transmission cycle and develop potential control methods. In the present study, we screened for possible interactions between virus, mosquito genotypes and microbiota. Based on our results, we discuss vector competence for DENV-2 in three geographically distinct *Ae. aegypti* populations, as well as variability in the immune response and in microbiota and support for multiple mechanisms underlying variation in mosquito susceptibility to the virus.

To evaluate vector competence, it is crucial to characterize the rates of infection, dissemination and transmission. In our study, despite the small sample sizes, the VDA population had significantly lower infection, dissemination rates and viral titer at 10 dpi compared to the Vero population. In contrast, despite the VDA population and CA population showing no statistical difference in viral titer at 10 dpi, the viral titer was significantly less at 2 and 3 dpi between the VDA and CA populations. Considering all the data collected, the VDA population revealed a relatively high refractoriness to DENV-2 compared to the other two populations (Table 1; Fig. 1). In contrast, although the CA population showed high rates of infection and dissemination, its transmission rate was relatively low. The Vero population had a similar susceptibility to DENV-2 as the CA population but a significantly higher transmission rate, suggesting that the Vero population is the better vector. However, due to the small sample sizes, these results should be viewed with caution.

Since the three geographically distinct mosquito populations represented different susceptibilities to and TE of DENV-2, we defined them as populations with unique backgrounds and thus they will be used to further investigate the potential factors involved [49]. Interestingly, the feeding rate of *Ae. aegypti* from this study was low, especially that of the VDA population, possibly due to relatively fewer years in colony from the field and slower adaptation to laboratory conditions [50].

The eclipse period early in the EIP indicates that virus titer drops in the mosquito during ingestion [48], which might be indicative of antiviral response in the mosquito, although this is speculative as the mechanism is not well understood. All three populations in this study exhibited an eclipse phase (Fig. 1b). Interestingly, the viral titer in the CA population remained similar between 1 and 2 dpi and then increased significantly at 3 dpi, reaching titers of > 40-fold and > 180-fold higher than those in the Vero and VDA populations, respectively.

The high viral titer in the CA population likely explains the upregulation of several immune-related transcripts at 3 dpi (Fig. 2). The NF-κB-like transcription factor Rel-1A and its co-activator Rel-1B are downstream factors in the Toll pathway, which receive activation signals to translocate into the nucleus to initiate transcription during infection with pathogens [51]. Both signal transduction genes were upregulated in the CA population at 3 dpi. Rel-1A gene transcription increased under DENV-2 infection in *Ae. aegypti* at 10 dpi [16] but not that of Rel-1B. However, temporal and population differences might cause the immune response to vary. Because the Toll pathway is associated with antiviral response in *Ae. aegypti* [16, 52, 53], increasing Rel-1 transcripts in the CA population might be a response to the higher viral titer. The Ago-2 transcript was also upregulated at 3 dpi in the CA population. The Ago-2 protein is the effector in the RNA-induced silencing complex (RISC) and uses a guide strand to target viral RNA for cleavage and degradation via the RNAi pathway [54, 55]. In *Ae. aegypti*, the silencing of Ago-2 gene expression decreased the incubation time of DENV and increased the transmission rate [11]. The RNAi biological response also limited the replication of CHIKV in *Ae. aegypti* [12] and Oŉyong’nyong virus in *Anopheles gambiae* [10], supporting the assertion that the RNAi pathway is one of the most important antiviral responses in mosquitoes. That the CA population did not show the highest titer at 10 dpi might suggest that both the Toll and RNAi pathways played antiviral roles once induced due to the high viral load at 3 dpi.

Gene transcripts of the apoptosis and autophagy pathways were altered at 3 dpi in both the CA and Vero populations. Apoptosis has been identified as an antiviral response in insects [56]. Moreover, previous studies showed that apoptosis pathway genes were highly expressed in a DENV-2 refractory strain of *Ae. aegypti* [57] and that silencing of the pro-apoptotic gene Dronc allowed the DENV-2 infection rate to increase [21]. Apoptosis activation limits ZIKV and DENV-2 proliferation in the midguts of *Ae. aegypti* [58]. Given the significant increase in Dronc expression observed in the CA population at 3 dpi, these findings might suggest that the high DENV-2 titer at the same time point for this population triggered an increase in the apoptosis pathway. However, our study also showed upregulation of the expression of IAP-1 in the CA population, suggesting possible negative feedback to the highly activated apoptosis pathway. The autophagy pathway has also been shown to serve an immunity function in insects [59]. The ATG5 gene product is an initiator of autophagosome formation [60, 61] and a key component in the autophagy pathway. Several ATG genes were upregulated under DENV infection in an *Ae. aegypti* refractory strain [22, 62], and autophagy activation decreased DENV-2 titer in a recent study [23]. However, the VDA population, which our data implicate as being refractory to DENV-2, did not exhibit significant changes in ATG5 expression at any time point, although the highest expression at 2 dpi corresponded to a low DENV-2 titer. Conversely, ATG5 expression in CA and Vero populations was significantly upregulated at 3 dpi, corresponding to time points at which mosquitoes from these populations had high DENV-2 titers. Our results, in combination with those from the literature, suggest that interactions between apoptosis, autophagy and DENV-2 are complex and warrant further investigation. Moreover, the apoptosis and autophagy pathway transcripts were altered in both CA and Vero populations, suggesting they might not be the major antiviral response, at least in the three populations we tested.

The IMD and JAK/STAT pathways have been previously characterized as antiviral pathways in insects [17–20]. In the present study, however, transcripts from neither pathway were upregulated in any of the three mosquito populations at any time point, while Rel-2 transcription was downregulated at 2 dpi in the Vero population. Although the CA population had increased transcription of genes in several pathways, the uniqueness of the involvement of the Toll and RNAi pathways in infection in the CA population was noteworthy. Taking into account that this population had high infection and dissemination rates but a relatively low transmission rate, both the Toll and RNAi pathways might play a role in SGIB and SGEB. Future studies are necessary to specifically test this hypothesis.

Surprisingly, the refractory population VDA did not show any significant differences in transcription of immune-related genes in the first three days of DENV-2 infection. We therefore compared the basal transcript levels of genes in the Toll pathway between populations after blood-feeding (Fig. 3). The Rel-1A and Rel-1B transcripts in the VDA population were highly expressed compared to their expression in the other two populations at 2 and 3 dpb, suggesting the possibility that blood triggered the Toll pathway in the VDA population without virus treatment. In addition to its antiviral function, Rel-1 has been implicated as an important factor in regulating the antifungal and antibacterial immune responses via the Toll pathway in *Ae. aegypti* [63–65]. In another study, a symbiotic bacterium induced reactive oxygen species through the Toll pathway in *Ae. aegypti* and limited DENV proliferation [66], indicating the possibility that microbiota influence vector competence indirectly by activating the Toll pathway [26].

Since the microbiota composition is known to change after blood-feeding [67, 68] and because in the present study the Rel-1A and Rel-1B transcripts were upregulated at 2 and 3 dpb in the VDA population, we focused on post-blood feeding differences in microbiota between populations. The microbiota screening revealed variation in microbiota in the VDA population but showed limited species abundance in the CA population (Fig. 4; Additional file 1: Tables S3–S7).

In the bacteria screening, *Asaia* (*Rhodospirillales*) sequences were identified in the VDA and Vero populations but not in the CA population. Members of genus *Asaia* are commonly present in different mosquito species [69, 70]. Bacteria in this genus are known to interact with resident microbiota [71, 72] and to influence vector metabolism [73]. In particular, *Asaia* was found to modulate midgut pH through glucose metabolism in the *Anopheles* vector, which in turn promotes* Plasmodium* microgametogenesis (i.e. development of male gametes), a critical step in the malaria-parasite life-cycle that when increased enhances infection of the mosquito midgut [74]. In contrast, *Asaia* was also reported to negatively impact* Plasmodium* by activating immune genes in two *Anopheles* species that triggered anti-plasmodium responses [75]. However, whether *Asaia* is involved in the response to DENV-2 infection in *Ae. aegypti* is still unclear.

*Elizabethkingia anophelis* (*Flavobacteriales*), a bacterium originally isolated from* Anopheles gambiae* [76] and commonly found in *Ae. aegypti* mosquitoes [77, 78] was recognized in the present study in all three population through Blast analyses. This bacterium was linked to the mitigation of iron stress in *An. gambiae* during blood-meal intake by digesting red blood cells [79]. *Elizabethkingia anopheles* was also linked to a decrease in ZIKV in infected *Aedes albopictus* mosquitoes, although the mechanism(s) underlying this negative interaction was not determined [80]. Even though *E. anophelis* was detected in all three populations in the present study, we were unable to determine whether this bacterium was involved in the interplay with dengue virus and *Ae. aegypti*.

Although to date, only a limited number of fungal species have been identified in *Ae. aegypti* [46, 70], some of the fungal genera we identified have been reported to occur across a broad range of different insects, including those in the order Diptera, such as *Drosophilids*, as well as social insects in the order Hymenoptera [81]. In the VDA population, two unique yeasts from the order *Saccharomycetales*, *S. etchellsii* and *R. colostri* were detected, along with the mycorrhizal fungus *Paraglomus occulatum* (*Paraglomeralles*) [82] and *P. allantivorans* (*Tremellales*), described as a parasite of other fungi [83]. The *M. guilliermondii* (*Saccharomycetales*), *T. anomala* (*Georgefischeriales*) and *W. canadensis* (*Wallemiales*) were shared among the VDA and Vero populations. Our data indicate that two fungal species, *C. zeylanoides* (*Saccharomycetales*) and *W. canadensis*, appeared in all three populations. The discovered species are classified in orders and subphyla of fungi that have already been identified in mosquitoes and their larval habitats through next-generation sequencing [84, 85]. Considering the geographical distance of the three populations that were investigated in the present study, it is possible that the species in common were acquired from the climate-controlled room in which the mosquitoes were reared [24]. However, some microbial members could be inherited from mother to larvae by attaching to the egg surface and, therefore, introduced to the new breeding site [19, 86, 87].

Two insect-specific flaviviruses,* Phlebotomus*-associated flavivirus and* Flaviviridae* sp. Arg/Fla/Aeae/02 isolate, were identified in the VDA population, while only* Phlebotomus*-associated flavivirus was detected in the Vero population. However, none of the flaviviruses were found in the CA population. Both strains were reported previously in different *Ae. aegypti* populations [88, 89]. Although we did not identify the same species, insect-specific flaviviruses have been shown to interact with the mosquito immune system and result in a significant reduction of arbovirus co-infection in vitro [90]. While microbiota have been shown to potentially effect vector competence [29, 35], evidence to connect virus infection in the mosquito with the species identified from the three populations is lacking.

## Conclusions

The results of the present study support the potential involvement of immune pathways and microbiota in shaping variation in vector competence for DENV-2 in *Ae. aegypti* and, despite the small sample number, add to existing knowledge by showing that antiviral response pathway activation and components of the microbiota differ by geographic origin of the mosquito population. Among the three geographically distinct *Ae. aegypti* populations studied, the CA population had a low transmission rate and might be associated with the upregulation of immune-related transcripts, especially the Toll and RNAi pathways. The competent Vero population had altered transcription of genes in the apoptosis and autophagy pathways, suggesting minor effects on antiviral functions as the titer was the highest among all three populations at 10 dpi. Moreover, the infection, dissemination and transmission rates were also higher in the Vero population than in the other two populations. Although the refractory VDA population did not show any transcriptional differences after infection, its Rel-1 basal level was higher than that in the other populations after blood ingestion, suggesting that the VDA population might have more efficient antiviral signaling. Additionally, the VDA population contained the most diverse microbiota species, which may contribute to its reduced vector competence. Deciphering the components of mosquito microbiota and targeting the microbial candidates with particular attributes could contribute towards reducing the mosquito-borne diseases through novel symbiotic control approaches [91–93]. Taken together, the present studyt covers several aspects that identify possible factors that influence resistance to DENV-2 in *Ae. aegypti* and provide information that will contribute towards potential new prospective control methods.

## Supplementary Information

Additional file 1: Table S1. Primer list. Table S2. The Rel-2 and Dome relative fold change in transcription between blood-fed Ae. aegypti populations. Table S3. Bacterial 16S rRNA library screening. Table S4. Bacterial isolates lineage. Table S5. Fungal 18S rRNA library screening. Table S6. Fungal isolates lineage. Table S7. Flavivirus Sequences.

## Data Availability

All data generated or analyzed during this study are included in this published article and its supplementary information files.
