# Supplementary material for: Immune-related transcripts, microbiota and vector competence differ in dengue-2 virus-infected geographically distinct Aedes aegypti populations
Source: Parasit Vectors. 2023 May 19;16:166. doi: 10.1186/s13071-023-05784-3 (PMC10199558; doi:10.1186/s13071-023-05784-3)
Supplement: Supplementary file 1 — Additional file 1: Table S1. Primer list. Table S2. The Rel-2 and Dome relative fold change in transcription between blood-fed Ae. aegypti populations. Table S3. Bacterial 16S rRNA library screening. Table S4. Bacterial isolates lineage. Table S5. Fungal 18S rRNA library screening. Table S6. Fungal isolates lineage. Table S7. Flavivirus Sequences. [file 13071_2023_5784_MOESM1_ESM.docx]

Additional file 1

**Table S1.** List of Primers.

| Gene | Forward Primer | Reverse Primer |
| --- | --- | --- |
| Rel-1A | **AGAAAAGCCATGTCCGATCTGGTGA** | **CCTGTTTGTGCACGTTGGTATGCT** |
| Rel-1B | **AAACTTCCTCTGCCTCCCAAA** | **TACGCATGGAACCCTTCCGAATGA** |
| Rel-2 | **GGACTGGGGTTCTTTCTCGG** | **ATTTGTCTCGTGGCCGGTAG** |
| Dome | **GCATCAGCGGGAAAGTTCCAATGT** | **AGCTTGTAATCGGTGGGAATCGG** |
| ATG5 | **CTCAGCTATCCGGACAACTTTC** | **GAACCGTCATCTCAAGCCTTAC** |
| Dronc | **TCGTGGTTTGTGGAGAGTATG** | **GTCCGATATGTGCTGGAATCT** |
| IAP-1 | **GTGAAACCCAAAGGCCAAAG** | **CTCGGATTGTCGGAACCTATAC** |
| Ago-2 | **ACAACAGCAACAATCCCAGA** | **GTGGACGTTGATCTTGTTGG** |
| S7 | **ACAAGAACCAGCAGACCAC** | **TCCGGGAATTCGAACGTAAC** |
| DENV2 | **CTWTCAATATGCTGAAACGCG** | **CGCCACACAAGGGCCATGAACAG** |
| Flavi JV2a | **AGYMGHGCCATHTGGTWCATGTGG** | **GTRTCCCADCCDGCDGTRTCATC** |
| Flavi JV2b | **AGCCGYGCCATHTGGTATATGTGG** | **GTRTCCCAKCCWGCTGTGTCGTC** |
| Flavi JV2c | **AGYCGMGCAATHTGGTACATGTGG** |  |
| Flavi JV2d | **AGTAGAGCTATATGGTACATGTGG** |  |
| 16srRNA | **AGAGTTTGATCATGGCTCAG** | **TACGGCTACCTTGTTACGACTT** |
| 18srRNA | **TAATTCTAGAGCTAATACATG** | **GGAAACGTCCTTGGCAAA** |

**Table S2.** The Rel-2 and Dome relative fold change in transcription between blood-fed *Ae. aegypti* populations.

| Gene | Vilas do Atlântico (VDA) | | | California (CA) | | | Vero | | |
| --- | --- | --- | --- | --- | --- | --- | --- | --- | --- |
|  | Day 1 | Day 2 | Day 3 | Day 1 | Day 2 | Day 3 | Day 1 | Day 2 | Day 3 |
| Rel-2 | 1.12±0.64 | 0.62±0.26 | 1.95±0.72 | 4.95±3.84 | 0.39±0.23 | 3.92±2.90 | 1.06±0.37 | 1.01±0.20 | 1.03±0.31 |
| Dome | 0.25±0.15 | 0.68±0.37 | 2.92±1.49 | 1.17±0.50 | 0.88±0.41 | 5.45±0.51 | 1.10±0.55 | 1.02±0.24 | 1.09±0.57 |

**Table S3.** Bacterial 16srRNA library screening.

| Location | Species | Blast results | |  | |  | |
| --- | --- | --- | --- | --- | --- | --- | --- |
|  |  | Accession Number | Identity [%] | | Query Cover [%] | |  |
| Vilas do Atlântico (VDA) | *Asaia bogorensis* | [NR_113849.1](https://www.ncbi.nlm.nih.gov/nucleotide/NR_113849.1?report=genbank&log$=nuclalign&blast_rank=1&RID=DNT3A7ND013) | | 98.07 | | 96 | |
|  | *Asaia platycodi* | [NR_112879.1](https://www.ncbi.nlm.nih.gov/nucleotide/NR_112879.1?report=genbank&log$=nuclalign&blast_rank=1&RID=DNVCPJNH013) | | 99.58 | | 99 | |
|  | *Elizabethkingia anophelis* | [NR_116021.1](https://www.ncbi.nlm.nih.gov/nucleotide/NR_116021.1?report=genbank&log$=nuclalign&blast_rank=1&RID=DNSZFPFD01R) | | 98.86 | | 100 | |
| Vero | *Asaia platycodi* | [NR_112879.1](https://www.ncbi.nlm.nih.gov/nucleotide/NR_112879.1?report=genbank&log$=nuclalign&blast_rank=1&RID=DNVCPJNH013) | | 99.43 | | 98 | |
|  | *Elizabethkingia anophelis* | [NR_116021.1](https://www.ncbi.nlm.nih.gov/nucleotide/NR_116021.1?report=genbank&log$=nuclalign&blast_rank=1&RID=DNSZFPFD01R) | | 95.32 | | 68 | |
| California (CA) | *Elizabethkingia anophelis* | [NR_116021.1](https://www.ncbi.nlm.nih.gov/nucleotide/NR_116021.1?report=genbank&log$=nuclalign&blast_rank=1&RID=DNSZFPFD01R) | | 98.28 | | 98 | |

**Table S4.** Bacterial isolates lineage (partial).

| BACTERIA |  |  |  |
| --- | --- | --- | --- |
| Species | **Order** | **Class** | **Phylum** |
| *Asaia bogorensis* | [Rhodospirillales](https://www.ncbi.nlm.nih.gov/Taxonomy/Browser/wwwtax.cgi?mode=Undef&id=204441&lvl=3&keep=1&srchmode=1&unlock) | Alphaproteobacteria | Proteobacteria |
| *Asaia platycodi* | [Rhodospirillales](https://www.ncbi.nlm.nih.gov/Taxonomy/Browser/wwwtax.cgi?mode=Undef&id=204441&lvl=3&keep=1&srchmode=1&unlock) | Alphaproteobacteria | Proteobacteria |
| *Elizabethkingia anophelis* | Flavobacteriales | Flavobacteriia | Bacteroidetes |

**Table S5.** Fungal 18srRNA library screening.

| LOCATION | SPECIES | BLAST RESULTS |  |  |
| --- | --- | --- | --- | --- |
|  |  | Accession Number | Identity [%] | Query Cover [%] |
| Vilas do Atlântico (VDA) | *Candida zeylanoides* | [NG_062651.1](https://www.ncbi.nlm.nih.gov/nucleotide/NG_062651.1?report=genbank&log$=nuclalign&blast_rank=1&RID=Y2DEYS89013) | 99.14 | 97 |
|  | *Meyerozyma guilliermondii* | [NG_063363.1](https://www.ncbi.nlm.nih.gov/nucleotide/NG_063363.1?report=genbank&log$=nuclalign&blast_rank=1&RID=Y4RZ27FJ016) | 99.63 | 96 |
|  | *Paraglomus occulatum* | [NG_017179.1](https://www.ncbi.nlm.nih.gov/nucleotide/NG_017179.1?report=genbank&log$=nuclalign&blast_rank=1&RID=DNZD75C2013) | 83.37 | 79 |
|  | *Pseudotremella allantoinivorans* | [NG_062937.1](https://www.ncbi.nlm.nih.gov/nucleotide/NG_062937.1?report=genbank&log$=nuclalign&blast_rank=1&RID=DNZ3W9Z3013) | 87.39 | 36 |
|  | *Rhodosporidiobolus colostri* | [NG_062179.1](https://www.ncbi.nlm.nih.gov/nucleotide/NG_062179.1?report=genbank&log$=nuclalign&blast_rank=1&RID=Y4S8B1YR013) | 99.08 | 96 |
|  | *Starmerella etchellsii* | [NG_063427.1](https://www.ncbi.nlm.nih.gov/nucleotide/NG_063427.1?report=genbank&log$=nuclalign&blast_rank=1&RID=Y2D73A9N013) | 97.56 | 96 |
|  | *Tilletiaria anomala* | [NG_061031.1](https://www.ncbi.nlm.nih.gov/nucleotide/NG_061031.1?report=genbank&log$=nuclalign&blast_rank=1&RID=Y20XMKUU013) | 83.98 | 83 |
|  | *Wallemia canadensis* | [NG_062401.1](https://www.ncbi.nlm.nih.gov/nucleotide/NG_062401.1?report=genbank&log$=nuclalign&blast_rank=1&RID=Y23BG4RJ016) | 99.51 | 98 |
| Vero | *Candida zeylanoides* | [NG_062651.1](https://www.ncbi.nlm.nih.gov/nucleotide/NG_062651.1?report=genbank&log$=nuclalign&blast_rank=1&RID=Y2DEYS89013) | 99.26 | 96 |
|  | *Meyerozyma guilliermondii* | [NG_063363.1](https://www.ncbi.nlm.nih.gov/nucleotide/NG_063363.1?report=genbank&log$=nuclalign&blast_rank=1&RID=Y4RZ27FJ016) | 99.88 | 99 |
|  | *Tilletiaria anomala* | [NG_061031.1](https://www.ncbi.nlm.nih.gov/nucleotide/NG_061031.1?report=genbank&log$=nuclalign&blast_rank=1&RID=Y20XMKUU013) | 88.25 | 42 |
|  | *Wallemia canadensis* | [NG_062401.1](https://www.ncbi.nlm.nih.gov/nucleotide/NG_062401.1?report=genbank&log$=nuclalign&blast_rank=1&RID=Y23BG4RJ016) | 99.63 | 99 |
| California (CA) | *Candida zeylanoides* | [NG_062651.1](https://www.ncbi.nlm.nih.gov/nucleotide/NG_062651.1?report=genbank&log$=nuclalign&blast_rank=1&RID=Y2DEYS89013) | 99.96 | 100 |
|  | *Tilletiaria anomala* | [NG_061031.1](https://www.ncbi.nlm.nih.gov/nucleotide/NG_061031.1?report=genbank&log$=nuclalign&blast_rank=1&RID=DP3JKMAN016) | 76.78 | 89 |
|  | *Wallemia canadensis* | [NG_062401.1](https://www.ncbi.nlm.nih.gov/nucleotide/NG_062401.1?report=genbank&log$=nuclalign&blast_rank=1&RID=Y23BG4RJ016) | 99.26 | 99 |

**Table S6.** Fungal isolates lineage (partial).

| FUNGI |  |  |  |  |  |
| --- | --- | --- | --- | --- | --- |
| Species | **Order** | **Class** |  | **Subphylum** | **Phylum** |
| *Candida zeylanoides* | [Saccharomycetales](https://www.ncbi.nlm.nih.gov/Taxonomy/Browser/wwwtax.cgi?mode=Undef&id=4892&lvl=3&keep=1&srchmode=1&unlock) | [Saccharomycetes](https://www.ncbi.nlm.nih.gov/Taxonomy/Browser/wwwtax.cgi?mode=Undef&id=147537&lvl=3&keep=1&srchmode=1&unlock) |  | Saccharomycotina | Ascomycota |
| *Meyerozyma guilliermondii* | [Saccharomycetales](https://www.ncbi.nlm.nih.gov/Taxonomy/Browser/wwwtax.cgi?mode=Undef&id=4892&lvl=3&keep=1&srchmode=1&unlock) | Saccharomycetes |  | Saccharomycotina | Ascomycota |
| *Paraglomus occulatum* | [Paraglomerales](https://www.ncbi.nlm.nih.gov/Taxonomy/Browser/wwwtax.cgi?mode=Undef&id=214508&lvl=3&keep=1&srchmode=1&unlock) | [Glomeromycetes](https://www.ncbi.nlm.nih.gov/Taxonomy/Browser/wwwtax.cgi?mode=Undef&id=214504&lvl=3&keep=1&srchmode=1&unlock) |  | Glomeromycotina | Mucoromycota |
| *Pseudotremella allantoinivorans* | Tremellales | Tremellomycetes |  | Agaricomycotina | Basidiomycota |
| *Rhodosporidiobolus colostri* | Sporidiobolales | Microbotryomycetes |  | Pucciniomycotina | Basidiomycota |
| *Starmerella etchellsii* | [Saccharomycetales](https://www.ncbi.nlm.nih.gov/Taxonomy/Browser/wwwtax.cgi?mode=Undef&id=4892&lvl=3&keep=1&srchmode=1&unlock) | [Saccharomycetes](https://www.ncbi.nlm.nih.gov/Taxonomy/Browser/wwwtax.cgi?mode=Undef&id=147537&lvl=3&keep=1&srchmode=1&unlock) |  | Saccharomycotina | Ascomycota |
| *Tilletiaria anomala* | Georgefischeriales | Exobasidiomycetes |  | Ustilaginomycotina | Basidiomycota |
| *Wallemia canadensis* | Wallemiales | Wallemiomycetes |  | Wallemiomycotina | Basidiomycota |

**Table S7.** Flavivirus Sequences.

| Location | Virus | BLAST RESULTS |  |  |
| --- | --- | --- | --- | --- |
|  |  | Accession Number | Identity [%] | Query Cover [%] |
| Vilas do Atlântico (VDA) | Flaviviridae sp. isolate Arg/Fla/Aeae/02 NS5 gene, partial cds | [MT863349.1](https://www.ncbi.nlm.nih.gov/nucleotide/MT863349.1?report=genbank&log$=nuclalign&blast_rank=1&RID=E3110YR1016) | 96.98 | 85 |
|  | Phlebotomus-associated flavivirus isolate SA-JD-JA-AK-16-9-A16 polyprotein, NS5 region, (POLY) gene, partial cds | MN294941.1 | 96.73 | 91 |
| Vero | Phlebotomus-associated flavivirus isolate SA-JD-JA-AK-16-9-A16 polyprotein, NS5 region, (POLY) gene, partial cds | MN294941.1 | 97.94 | 81 |
| California (CA) | None Flavivirus detected |  |  |  |
